# Editorial: Digital Interventions in Mental Health: Current Status and Future Directions

**DOI:** 10.3389/fpsyt.2020.00111

**Published:** 2020-02-27

**Authors:** Elias Aboujaoude, Lina Gega, Michelle B. Parish, Donald M. Hilty

**Affiliations:** ^1^Department of Psychiatry and Behavioral Sciences, Stanford University, Stanford, CA, United States; ^2^Department of Health Sciences & Hull York Medicine School, University of York, York, United Kingdom; ^3^Department of Psychiatry and Behavioral Sciences, University of California, Davis, Davis, CA, United States

**Keywords:** telemedicine, digital mental health interventions, virtual reality therapy, artificial intelligence, computerized cognitive behavior therapy, telepsychiatry, electronic health record - (EHR), internet addiction

An honest supply-and-demand assessment of mental health services in 2020 leads one to conclude that treatment needs would be impossible to meet without increased leveraging of technology. Several inherent factors make mental health interventions particularly well suited to digital platforms: pronounced provider shortages; reduced reliance on examinations and testing; the stigma still attached to mental illness; and diagnosis-specific obstacles to visiting mental health clinics (1). As such, various technology-enabled platforms have been tested to support mental health treatment delivery, from internet-mediated video-based psychotherapy to virtual reality (VR) and artificial intelligence (AI) enabled programs. Yet the reach of digital mental health interventions (DMHIs) still falls short of its touted potential (2).

Internet-delivered cognitive behavioral therapy (ICBT) may be among the best-studied DMHIs (3). It has been used for nearly 20 years, during which it has been subjected to several efficacy trials. To determine more reliable response rates, Andersson et al. conducted an individual patient data meta-analysis, comprising 29 Swedish trials enrolling 2866 participants, and covering three categories of conditions: anxiety disorders, depression, and other. Overall, 65.6% of all clients receiving ICBT responded, and about a third achieved remission. More symptoms and female sex increased the likelihood of improvement, and having an anxiety disorder seemed to decrease it.

Children, adolescents, and young adults have received particular attention with respect to DMHIs. As “digital natives,” might they engage with, and benefit from, technology-enabled treatment more than those who did not grow up with technology? Garrido et al.'s meta-analysis focusing on young people with anxiety and depression suggests that DMHIs—in particular supervised DMHIs rather than standalone self-help—outperform “no intervention” but do no better than active alternatives (e.g., face-to-face therapy). Surprisingly, adherence of young people with DMHIs was generally low, mirroring reviews on DMHIs in adults (4, 5).

Other sub-populations that have received research attention include army veterans, older adults, and patients in Latin America. Boykin et al. examined the use of video-based therapy in the naturalistic setting of a clinic serving US veterans with post-traumatic stress disorder. In 74 veterans receiving at least one session of video-delivered cognitive processing therapy (CPT) or prolonged exposure (PE), the completion rate was higher for CPT, but attrition by session 7 was 50% (similar to in-person treatment in this population).

Among older adults, the use of DMHIs comes with unique obstacles and advantages. Seifert et al. dissect those in an opinion article that focuses on healthcare inequalities and how, depending on awareness, training and public health priorities, new technologies can serve to either mitigate or magnify the disparities that already exist across the age spectrum.

As a region, Latin America suffers from unique access-to-care challenges, but the territory also enjoys relatively good internet and smartphone penetrance (6), making DMHIs a possible solution. In a scoping review of 22 studies that cover prevention, treatment, education, and symptom self-management, Jiménez-Molina et al. explored the potential for leveraging internet-based interventions in mental health in Latin America. Results from the three RCTs identified were mixed, and, while most feasibility and pilot studies showed reasonable acceptability, participant retention was challenging, follow-ups were short, and data on costs and outcomes were limited. The authors conclude that more evidence is required before DMHIs can be considered a realistic remedy to access and delivery problems in Latin America.

But DMHIs do not have to replace traditional care altogether; they can also play a role alongside or preceding traditional treatment. In a UK study, Duffy et al. tested a stepped care model for treating 124 patients with severe depression and anxiety using ICBT as a prequel for a high-intensity face-to-face intervention. Significant reductions were noted across primary outcome measures from baseline to ICBT treatment exit, and from ICBT exit to service exit. Results support the use of ICBT as a means to reduce frustrating waiting times and enhance efficiency.

Another example of DMHIs working “with” traditional treatments may be the use of assistive technologies to address specific deficits within a disorder that contribute to functional disability. In a multinational study involving 243 participants, Cerga-Pashoja et al. tested an open-source text simplification tool designed to help people with autism spectrum disorder better comprehend complex texts, metaphors and idioms—a common challenge in this condition. The tool significantly enhanced functioning.

The future of technology-enabled treatment in mental health is probably best captured in the VR and AI “revolutions” unfolding within the larger space of DMHIs. VR has been tested for its possible therapeutic value in mental health for almost as long as it has been exploited for gaming purposes—a quarter century (7)—but research into VR treatments has accelerated considerably in recent years, particularly for anxiety disorders. Yet VR's reach remains limited among patients and therapists (2), a fact that Boeldt et al. dissect in their opinion piece, suggesting educational, practice-based and research steps to take full advantage of what VR therapeutics have to offer.

AI and machine learning are seen as transforming fields like radiology and pathology (8, 9), and, in medicine, more broadly, they hold the promise of bringing individualized low-cost interventions that can be easily scaled. But what is their potential niche and pitfalls in mental health, specifically? In their viewpoint article, Miner et al. discuss how conversational AI may impact psychological and psychiatric care at the level of diagnosis, information gathering and treatment, and propose four possible approaches as guideposts that inform future research and policy.

Mental health interventions, digital or not, have to be documented in patient records held by clinical professionals and health services. In their opinion article, Strudwick et al. argue for opening up these records to patients on the grounds of empowerment and autonomy. The electronic health record now in common use greatly facilitates this process, yet patient portals still often limit access to mental health notes for reasons and controversies elucidated by the authors.

Finally, DMHIs represent potentially beneficial uses of technology in mental health. A parallel and similarly rich body of research has focused on the negative aspects of technology, including addiction, gaming, cyberbullying, and online impulsivity. However, these two areas of scholarship have grown in mutually insular ways with little cross-fertilization, despite some shared commonalities that Aboujaoude and Gega explore in a perspective piece. Collaboration between researchers in both camps is essential if we are to reach a more complete understanding of the issues lying at the technology-psychology intersection.

Overall, the studies and articles included in this special issue suggest that outcomes with DMHIs are comparable with traditional offerings; that some under-researched populations are receiving much needed attention; and that enthusiastic interest animates clinicians and industry professionals to apply the latest digital developments, including VR and AI, to mental health diagnosis, symptom-tracking and treatment. However, plenty remains to be done to address some basic shortcomings that are borne out in the present issue, including: adherence and engagement challenges; access to technology obstacles; lack of cost effectiveness data; lack of long-term research; and the paradoxical process by which digital tools can sometimes serve to fortify rather than diminish healthcare inequalities. Given the relatively capped supply of traditional mental health treatments, and the ethical imperative to meet the ever-increasing demand, DMHIs are likely to be an unavoidable part of any solution to treatment access issues. This special issue highlights the dizzying diversity and richness propelling the field of DMHIs as well as the limitations still holding it back. Further rigorous, yet pragmatic, research—across platforms, populations, diagnoses and interventions—is needed to arrive at a more realistic assessment of the true potential of DMHIs.

## Author Contributions

EA, LG, MP, and DH contributed to the design, review and editing of the Research Topic and to the editorial summarizing it.

## Conflict of Interest

The authors declare that the research was conducted in the absence of any commercial or financial relationships that could be construed as a potential conflict of interest.
